# Anti-pan-neurofascin IgG3: insights about an emerging autoimmune nodoparanodopathy

**DOI:** 10.1055/s-0045-1812035

**Published:** 2025-10-15

**Authors:** Gabriel Erzinger, Mayra Emi Guinoza Inushi, Laura Fiuza Parolin, Gabriel de Deus Vieira, Marcus Vinícius Magno Gonçalves

**Affiliations:** 1 Universidade da Região de Joinville, Departamento de Medicina, Joinville SC, Brazil.

**Keywords:** Polyneuropathies, Autoantibodies, Immunoglobulin G

## Abstract

Neurofascin constitutes a family of cell-surface proteins identified more than 4 decades ago, produced through alternative RNA splicing, with various isoforms expressed in neural tissues. With the emergence of chronic inflammatory demyelinating polyneuropathy (CIDP) subtypes characterized by distinct pathological mechanisms, antineurofascin antibody-mediated neuropathies have gained attention and are now categorized as autoimmune nodoparanodopathies. Among these, the anti-pan-neurofascin immunoglobulin G3 (IgG3) subtype presents a particularly severe and diagnostically-challenging phenotype, marked by a fulminant clinical course, diverse symptomatology, and high rates of morbidity and mortality. Despite its clinical relevance, to date, no comprehensive review has focused specifically on this manifestation, highlighting a significant gap in the literature. To address this, we herein review the seven reported cases and explore the proposed pathophysiological mechanism involving the destruction of the node of Ranvier via hyperactivation of membrane attack complex (MAC) formation. Additionally, we examine emerging evidence supporting the use of eculizumab as a potential therapeutic option, alongside other treatment strategies. Finally, we discuss the role of standardized antibody assays, serological analyses, and neurophysiological studies in improving diagnostic accuracy.

## INTRODUCTION

In 2013, Uncini et al. 1 proposed a new classification for dysfunctions of the node of Ranvier, termed *nodoparanodopathy* , initially described in antiganglioside antibody-mediated neuropathies. This classification differs from the traditional demyelinating and axonal classifications, focusing on the specific site of action, such as the node, paranode, juxtaparanode, or internode regions. The previous classification divides polyneuropathies into two groups: the first affects the myelin/Schwann cells, and the second affects the axon, which often leads to imprecise diagnoses in some electrophysiological findings, particularly in Guillain-Barré syndrome (GBS) subtypes. This paradigm shift is significant as it provides a more precise correlation between clinical presentation and underlying pathophysiological mechanisms, challenging the notion that the nodal region is the sole pathological target and the misconception of a universally-poor prognosis in such diseases. 1 Furthemore, in those regions. an antibody target of relevance to the clinical practice is the neurofascin (NF) family of proteins, first described in 1987 in chicks. These neural surface proteins have several isoforms, including NF140, NF155, NF166, NF180, and NF186, each associated with specific regions of the nodal/paranodal segment. 2 3 Moreover, they have alternative expressions at different times between isoforms, with their formation occurring through an alternative RNA splicing sequence, spanning from immature to mature neurons, with multiple different functions, to stabilization of paranodes and clustering voltage-gated sodium (Nav) channels, with all of them sharing the six immunoglobulin-like domains and a transmembrane region, but differing in the composition of their five possible fibronectin domains. 4 5

In 2021, the European Academy of Neurology (EAN) and the Peripheral Nerve Society 6 (PNS) updated their guidelines for the diagnosis and treatment of chronic inflammatory demyelinating polyneuropathy (CIDP), introducing a new classification known as *autoimmune nodopathies* . This designation was established to account for patients who met the 2010 European Federation of Neurological Societies (EFNS)/PNS criteria for CIDP but exhibited distinct clinical features and differing responses to treatment. Within this group (which encompasses antiNF-associated disorders), autoantibodies have been identified against three main NF isoforms: NF155, NF186, and NF140. 6 The NF155 isoform is located in the paranodal region and is typically associated with immunoglobulin G4 (IgG4), whereas the NF186 and NF140 isoforms, associated with IgG3 or IgG4, are found in the nodal region. Furthermore, both antiNF diseases have an acute or subacute onset. In some cases, NF186 and NF140 differ from NF155 due to their association with nephrotic syndrome. Additionally, their treatment profile is characterized by a better response to IgG therapy and the absence of central nervous system (CNS) involvement. 7

Moreover, in the group of autoimmune nodoparanodopathies, which also includes antibodies against contactin-associated protein 1 (Caspr1) and cell-adhesion molecules (contactin-1 [CNTN1]), one presentation that has recently been the subject of study is pan-antineurofascin, with a predominance of IgG3 which is noted for its explosive and confusing clinical presentation, with few cases reported in the literature. 6 8 In this context, given the recent knowledge of the disease and its clinical consequences, we decided to provide insights and describe the main findings related to anti-pan-NF IgG3 disease, including its pathophysiology, clinical manifestations, and diagnosis.

## PATHOPHYSIOLOGY

There are 4 IgG subclasses (IgG1, IgG2, IgG3, IgG4), which are ranked by their serum distribution in the body. They differ from each other due to distinct encoded regions, which result in varying half-lives, fragment crystallizable (Fc) regions, and functions. 9 Briefly, in the clinical practice, IgG1, the most common subclass, is associated with recurrent infections in the context of deficiency and, similarly to IgG3, responds to protein antigens. 10 Immunoglobulin G2 plays a role in the protection against capsular polysaccharide antigens, such as *Haemophilus* and *Pneumococcus* . 11 Immunoglobulin G4, which primarily responds to polysaccharide antigens, is associated with various diseases across nearly all medical specialties, particularly in IgG4-related diseases (IgG4-RDs). 12 Additionally, it plays a role in some autoimmune neurological diseases, including anti-IgLON5, anti-DPPX, MuSK, LGI1, and CASPR2-associated syndromes. 13 Moreover, each of the IgG subclasses has been identified as a predominant subgroup in cases of anti-pan-NF antibodies. 4

Among the four subclasses, IgG3 is the primary antibody that targets and binds to multiple NF isoforms in the anti-pan-NF IgG3 disease. It is an Ig subclass that represents 4 to 8% of the total IgG in the serum, and it is known for its potent proinflammatory properties, with a long and semirigid hinge region, favoring the binding to FcγR and C1q. Its half.-life is a controversial subject, as it is typically shorter but has recently been found to be similar to that of IgG1 in some cases, and it is a less studied subclass due to its structural and functional heterogeneity, being a candidate for future monoclonal antibody therapies. 10 14 15 Furthemore, the antibodies against pan-NF access the nodal and paranodal location by binding in the six Ig-like domains. 16 Its potential role in causing the explosive symptomatology results from a strong effector function, leading to the binding of complement C1q in the classical pathway demonstrated by Appeltshauser et al., is by his strong effector function, leading to the binding of complement C1q in the classic pathway demonstrated by Appeltshauser et al., 17 providing in-vitro and in-vivo evidence of complement-related pathology and acute axonal damage. Moreover, following this cascade, with the common pathway complement progression, C5-convertase cleaves the C5, forming the well-know membrane attack complex (MAC) by the association of C5b with C6 and C7 in interaction with C8, binding with several units of C9, which penetrates the lipid bilayer and lyses the cell with the creation of a pore. By multiple disulfide bridges, IgG3 autoantibodies are likely involved in hyperactivation, leading to MAC formation. 15 18

**Figure 1:**
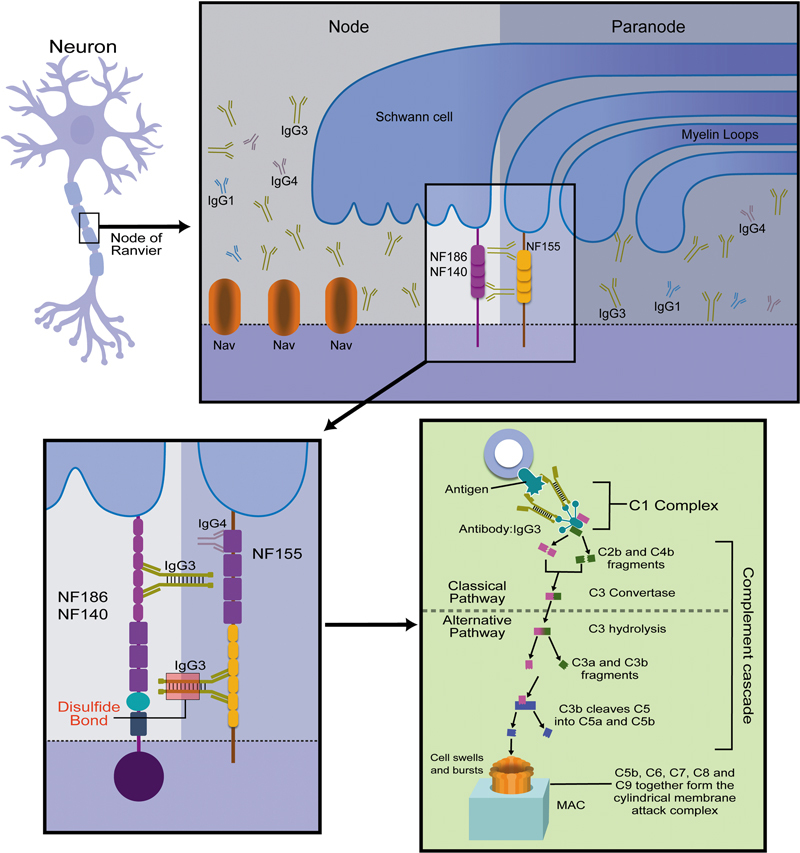
Pathophysiological mechanism involving anti-pan-neurofascin immunoglobulin G3 (IgG3) disease. The process illustrates the targeting of neurofascin 155 (NF155), which is located at the paranode, and of NF186/140, which is at the node, by IgG3, binding in their immunoglobulin domain. This interaction activates C1q, initiating the formation of the complement cascade and leading to the formation of the membrane attack complex (MAC).

**Figure 2:**
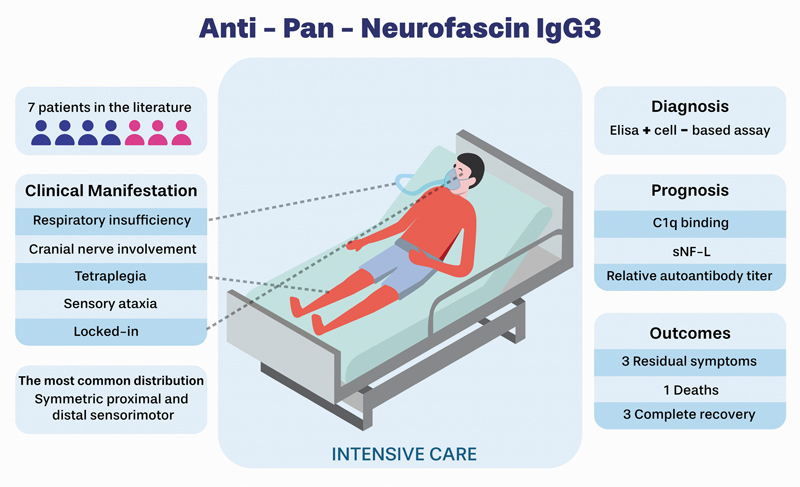
Key features of pan-anti-NF IgG3 disease: clinical characteristics, diagnostic distribution, prognostic assessment, and outcomes.

The possible pathophysiology process is represented in Figure 1 , and the key features of anti-pan-NF IgG3 disease are shown in Figure 2 .

## CLINICAL MANIFESTATIONS

The neuropathy associated with anti-pan-NF IgG3 typically presents with a fulminant onset. The clinical picture is not well known; it is often severe, presenting with tetraplegia, sensory and cranial nerve dysfunction, and almost locked-in syndrome. Multiple epitopes on the antibodies may play a role in determining the severity of the neuropathy. 7 12 14 There are reports 8 16 17 19 20 of severe muscle weakness, sensory ataxia, and autonomic dysfunction, which includes heartbeat and blood pressure instability, as well as paralytic ileus, reflecting significant impairment of the central and peripheral nervous systems. Furthermore, symptoms such as neuropathic pain, swallowing difficulties, and respiratory insufficiency may occur, resembling bulbar syndromes. 8 16 17 19 20

In the cases that we have found in the literature 8 16 17 19 20 ( Table 1 ), autoimmune neuropathy (AN) might be more common in patients aged between 50 and 74 years and predominantly in male subjects. In comparison, anti-NF155 IgG4 patients commonly presented onset at a young age and benign symptoms such as tremors and no severe motor dysfunctions, which did not occur in anti-pan-NF IgG3 patients. 16 17 Some patients with predominant IgG3 antibodies present with a clinical phenotype resembling GBS, characterized by ascending, symmetric proximal, and distal sensorimotor weakness. Although intravenous Ig (IVIg) may lead to initial improvement, these patients often experience a fulminant relapse within days or weeks, progressing to tetraparesis and respiratory failure. In other patients, the disease already begins with an immobilizing neuropathy requiring intermediate/intensive unit care, and they might need mechanical ventilation for months. Moreover, the disease presents high morbidity, but as a monophasic and possible reversible disorder. 4 17

**Table 1:** Clinical and laboratory characteristics of individuals with pan-anti-neurofascin immunoglobulin G3 (IgG3) disease

Study	(Stengel et al. 8 + Appeltshauser et al. 17 + Rohrbacher et al. 19 )*					Delmont et al. 16 *	Vallat et al. 20
Patient number	**1**	**4**	**6**	**7**	**11**	**CIDP-5**	**-**
Age (years)	71	72	63	52	74	50	70s
Sex	Male	Female	Male	Male	Female	Female	Male
Antibody predominant subclass	IgG3 > IgG2	IgG3 > IgG1	IgG3 > IgG4	IgG3 > IgG4 > IgG2	IgG3	IgG3 > IgG4	IgG3
Concomitant/Previous disease	DM	No	Respiratory infection, DM and cervical stenosis with myelopathy	DM	Respiratory infection and DM	No	Low-grade IgA lamda myeloma
Serum titer at first serum assessment	1:4,000	1:100	1:1,000	1:1,000	1:300	1:7,000	1:8,000
Distribution	Symmetric proximal and distal sensorimotor	Symmetric proximal and distal sensorimotor	Symmetric proximal and distal sensorimotor	Symmetric proximal and distal sensorimotor	Symmetric distal sensorimotor	Asymmetric distal sensorimotor ^‡^	Symmetric proximal motor and distal sensory
Clinical manifestation	Almost complete CN dysfunction, locked-in; respiratory insufficiency	Almost complete CN dysfunction, locked-in; respiratory insufficiency; neuropathic pain; pandysautonomia	Almost complete CN dysfunction, locked-in; respiratory insufficiency; sensory ataxia; severe blood pressure instability, paralytic ileus; cauda equina enhancement	CN III, V, and VI palsy, dysarthria, dysphagia; respiratory insufficiency; heartbeat and blood pressure instability; cauda equina enhancement	Dysphagia; sensory ataxia; reduced heart rate variability	Subacute onset with a progressive severe neuropathy; sensory ataxia; CN involvement; respiratory failure	Acute onset, proximal motor weakness of the four limbs, generalized areflexia, distal loss of pinprick and vibration sensations in the four limbs, mild respiratory failure, and late tetraplegia
Initial treatment response	IVIg, PEX, and RTX = none	IVIg and PEX = good; Ccs = none	Ccs = None	IVIg and PEX = good; Ccs = none	Ccs = good	PEX = good	IVIg = good
Final treatment response	−	RTX = good	IVIg and PEX = good	RTX = good	−	PEX and Ccs = good	IVIg and Ccs = none
Intensive care	Yes	Yes	Yes	Yes	Yes	Yes	Yes
Outcome	Death due to sepsis	Residual distal paresis of all extremities	Residual paresis of all extremities	Remissions with slight residues	Complete recovery**	Complete recovery**	Complete recovery**

Abbreviations: CIDP, chronic inflammatory demyelinating polyneuropathy; CN, cranial nerve; Ccs, corticosteroids; DM, diabetes mellitus; PEX, plasma exchange; RTX, rituximab; IVIg, intravenous immunoglobulin.

Notes: ^‡^ Evolved into symmetric proximal and distal distribution. *The studies include populations from the same cohort. The studies include populations from the same cohort, with data extracted from the supplementary material of the Appeltshauser study. 17 **Complete recovery is defined as a recovery without any symptoms.

## DIAGNOSIS

Certain clinical features are shared with GBS, but the immunopathogenic mechanisms are specific, primarily involving nodal/paranodal antibodies. Patients exhibiting a GBS-like onset and experiencing a relapse after initial treatment or a fulminant course of the disease (< 4 weeks) should be suspected of having AN. The symptoms are nonspecific, and they may include a monophasic severe disease presentation, partially locked-in syndrome, tetraplegia, normally symmetric sensorimotor weakness, cranial nerve dysfunction, autonomic instability, and respiratory failure. 4 17 21

Screening for IgG3 autoantibodies against NF isoforms is essential to guide targeted treatment strategies. Serological testing is advised through enzyme-linked immunosorbent assay (ELISA) and/or CBAs, which may be supported by tissue-based methods such as teased nerve fiber binding; however, these are not required for routine diagnosis. The CBAs have demonstrated higher sensitivity for IgG3 subclass detection compared with ELISA (13 versus 10 positive samples), interassay concordance was significantly higher between two independent ELISA runs (0.89) than between ELISA and CBA (0.64; *p* = 0.002). Nonetheless, IgG3 subclass detection exhibited low reproducibility (∼ 50%) in blinded testing, regardless of the method used, underscoring that test performance depends heavily on assay standardization, result interpretation, and laboratory expertise. 17 22 Furthermore, the access to ELISA and CBAs—primarily CBAs—is often limited, especially in resource-constrained settings, highlighting the need for broader availability and cost reduction. We recommend that patients with suspected autoimmune nodoparanodopathy be referred to specialized centers or, alternatively, that biological samples be forwarded to accredited laboratories capable of performing these assays. 4 22 Importantly, neither ELISA nor CBA should be considered prerequisites for initiating treatment, especially in severe and rapidly-progressive disease phenotypes. 4 17

Anti-NF autoantibodies, especially of the IgG3 subclass, can lead to complement activation and, consequently, axonal damage. Therefore, the C1q binding assay may help identify patients who not only have antibodies but also have an active pathological mechanism that contributes to disease severity. Furthermore, Appeltshauser et al. 17 found that serum neurofilament light chain (sNF-L) levels are highly increased in patients with anti-pan-NF compared with healthy patients (446.7 ± 433.4 versus 16.1 ± 6.2 pg/mL; *p* < 0.0001 and 0.0055 respectively). We can conclude that high levels of sNF-L, complement C1q binding, and relative autoantibody titer are directly correlated to the clinical severity of the disease. Although they cannot be used for diagnosis, they are valid for follow-up as biomarkers of disease activity, and they can assist the medical team in considering anti-pan-NF IgG3 as a possible diagnosis. 17

In additional paraclinical investigations, some findings are infrequent and nonspecific, such as enlargement of the nerve cross-sectional area, slowed nerve conduction, and, in some acute cases, non-excitable nerves. Sural nerve biopsy differentiates CIDP from AN, as it normally shows no signs of segmental demyelination or inflammation, but it does indicate axonal loss and paranodal elongation. Furthermore, cerebrospinal fluid (CSF) protein levels may be normal within the first 14 days, but they can increase significantly over time; antibody titers in CSF have not been systematically studied and, regarding autoimmune nodoparanodopathy, there is currently no evidence supporting significant intrathecal antibody production. Additionally, magnetic resonance imaging (MRI) scans showing cauda equina enhancement occurs more frequently than in NF155 neuropathy or CIDP. Other tests may be necessary according to the clinical presentation. 4 16

A comprehensive analysis of clinical, neurophysiological, and serological (when available) data are essential to establish a differential diagnosis from other conditions. Among the diseases, GBS, CIDP and others, AN should be considered. 4 22 Finally, further studies are necessary to enhance diagnostic precision and improve clinical outcomes in this patient population.

## TREATMENT

We have searched the literature on anti-pan-NF IgG3, 8 16 17 19 20 and the treatment prescribed and the clinical outcomes are presented in Table 1 . In these studies, 8 16 17 19 20 the treatment is not accurate and definitive, since it is based on case reports, and four major treatments are explored: corticosteroids (CCs), plasmapheresis (PEX), rituximab (RTX), and IVIg. Among the seven cases presented, two did not exhibit clinical improvement following the proposed treatment interventions. In one of these cases, 20 there seemed to be a natural history of the disease associated with a gradual improvement regardless of the therapeutic measures. Furthermore, of the four therapies used, the antibody to IVIg and PEX provides the same amount of recovery. We will address the therapies separately with their mechanisms of action and current evidence.

Corticosteroids are a well-known therapy used in almost all medical specialties, such as rheumatology, neurology and dermatology, with broad use in autoimmune conditions. Its mechanism of action is extensive, involving genomic and nongenomic pathways. 23 The most recognized pathway operates through a decrease in the formation of arachidonic acid derivatives via the promotion of lipocortin-A synthesis, which inhibits phospholipase A2, thereby promoting an anti-inflammatory effect. 18 Among the patients analyzed, 8 16 17 19 20 glucocorticoids were the therapy with the highest rate of ineffectiveness, with 4 cases showing no clinical response and 2 cases showing a response, though with CCs only as an adjunctive therapy, except for patient 11 (Table 1; the studies by Stengel et al., 8 Appeltshauser et al., 17 and Rohrbacher et al. 19 included populations from the same cohort). According to the guidelines for patients with CIDP, 6 one criterion that may lead to the suspicion of nodoparanodopathy is resistance to treatment with glucocorticoids. In such cases, alternative treatments may be more appropriate, as Ccs have a minimal impact on B cell function and Ig production. 24

Plasmapheresis is an extracorporeal treatment based on the exclusion, return or removal of plasma components by centrifugation or filtration using semipermeable membranes. 25 In anti-pan-NF patients, among the 6 instances of this treatment, 4 resulted in clinical improvement, even without complete remission, which seems to reflect in the final outcome. As it is a condition with higher titers of autoantibodies with a fulminant course, based on the pathophysiology, early therapy could yield better outcomes, such as shortened disease duration and the prevention of axonal damage, with our group in accordance with the idea of PEX as a first therapy in patients with Ig3 antibody disease. 4

Rituximab is an anti-CD20 monoclonal antibody selective to B cells with no plasma cell activity, and it is an actual treatment option for patients with IgG4 antibody diseases and recommended as an off-label option by the nodoparanodopathies guidelines. 4 6 26 The precise role and function of CD20 remain as an enigma, with various hypotheses regarding its mechanism of action and differing contributions to the clinical effects observed regarding anti-CD20 therapies. 27 In the literature, 8 16 17 19 20 three patients have undergone this treatment, with two showing documented clinical improvement. Different RTX protocols were used in these cases, including 1 or 2 dose regimens, dosages ranging from 855 mg to 1,000 mg, and intervals of 1 or 2 administrations within 2 weeks. Future studies are needed to standardize the dosing protocols and better assess the true therapeutic efficacy of RTX in this population. Beyond that, a recent study hypothesized that the ineffectiveness of this treatment in this population is attributed to its inability to directly target plasmablasts or long-lived plasma cells, indicating that anti-pan-NF antibody-associated neuropathy would be a plasmablast-mediated disease. 19 28

Intravenous Ig is a concentrate of pooled Igs, with IgG comprising more than 90% of the preparation; its role in the common pathway is controversial and highly complex. As a possible mechanism, it stimulates the inactivation of C3 convertase precursors at high doses and reverses this function at lower doses, providing proinflammatory or anti-inflammatory effects. 29 30 The lack of response to IVIg observed in previous studies could be explained by the production of high-titer antibodies with a fulminant case, which only results in a brief, limited temporal effect. Previous studies, 8 17 19 20 26 31 which reported treatment failure in another nodoparanodopathy, NF155, may support this explanation; however, the mechanism of action of IgG4 differs in that it does not bind to C1q.

### Future possibilities (complement-based treatment)

One treatment that could be a future response to anti-pan-NF IgG3 is eculizumab, whose mechanism of action is to bind to C5 and prevent its cleavage into anaphylatoxin (C5a) and C5b, reducing the formation of the terminal C5b-9 complex. 32 Based on the physiological pathway of IgG3, previous studies (cite 17 33 34 ) of complement-mediated pathology in IgG1/3 acetylcholine receptor antibody -seropositive myasthenia gravis (MG) demonstrated the superiority of anti-C5 over placebo, showing reduced hospitalization time and fewer disease exacerbations, with a well-tolerated safety profile. Morever, a prespecified subgroup analysis further reported better outcomes in patients receiving chronic IVIg. 34 Furthermore, another study that magnifies this hypothesis was the Japanese Eculizumab Trial for GBS, 35 a phase 2-trial that compared eculizumab and placebo in patients with severe GBS, exploring the use in the context of antibodies against gangliosides, leading to complement activation with deposition of MAC in Schwann cell membranes. The results favored motor function in some of the secondary endpoints; however, the trial was not designed for statistical comparisons between the groups.

Other complement-based treatments that could be a future response to anti-pan-NF IgG3 are currently under study for CIDP, and in pre/clinical stage for other diseases, such as: GL-2045 (Stradomer, Gliknik Inc./Pfizer Inc.), a recombinant Fc that binds to C1q, riliprubart, a humanized anti-C1s MAb that inhibits the classic complement pathway, zilucoplan, a cyclic peptide which binds to complement C5 and inhibits its cleavage into C5a and C5b, and ravulizumab, an eculizumab biosimilar. 26

In conclusion, anti-pan-NF IgG3 is an autoimmune nodoparanodopathy characterized by a fulminant course that often requires intensive care. Its pathophysiology involves complement-mediated attacks on the nodal/paranodal regions through the formation of the MAC complex. Immunoglobulin G3 autoantibodies, by multiple disulfide bridges, are likely involved in hyperactivation, leading to MAC formation. Clinical assessment, as well as neurophysiological and serological analyses, are essential to establish a diagnosis. Future research is needed to standardize antibody assay protocols and enhance diagnostic reliability across laboratories. Furthermore, the clinical phenotypes are marked by a long course of GBS symptoms or fulminant symptoms. Finally, patients with this disease require more studies supporting effective treatments, thereby making therapeutic approaches less uncertain; morever, complement-based therapies offer promising options for future treatment options.
